# Long-term trends in international medical electives fees: a database mining study

**DOI:** 10.1186/s12909-024-05123-9

**Published:** 2024-02-19

**Authors:** Kai Beckschulte, Ann-Kathrin Lederer, Maximilian Andreas Storz

**Affiliations:** 1 Department of Internal Medicine II, Centre for Complementary Medicine, Medical Center – University of Freiburg, Faculty of Medicine, University of Freiburg, Freiburg im Breisgau, Germany; 2 Department of General, Visceral and Transplant Surgery, University Medical Centre of the Johannes Gutenberg University, Mainz, Germany

**Keywords:** Abroad elective, International medical elective, Global health, Internationalization, Fees, Medical education, Medical ethics, International health care, Undergraduate education

## Abstract

**Background:**

Abroad medical electives are recognized as high-impact practice and considered a necessity to provide global health training. As of recently, the COVID-19 pandemic and its related travel restrictions prohibited most international elective activities. Another important barrier to abroad electives that received comparably little attention is elective and application fees, which – combined – may be as high as $5000 per month, and may prevent students with limited financial resources from applying for an international elective. Elective fees have never been systematically analyzed and trends in teaching and application fees have rarely been subject to dedicated scientific investigations.

**Methods:**

Using data from two large elective reports databases, the authors addressed this gap in the literature. The authors analyzed trends in abroad elective fees within the last 15 years in some of the most popular Anglo-American elective destinations among students from Germany, including the United States of America, Australia, New Zealand, the Republic of South Africa, Ireland and the United Kingdom.

**Results:**

The authors identified *n* = 726 overseas elective reports that were uploaded between 2006 and 2020, of which *n* = 438 testimonies met the inclusion criteria. The United Kingdom and Australia were the most popular elective destinations (*n* = 123 and *n* = 113, respectively), followed by the Republic of South Africa (*n* = 104) and the United States of America (*n* = 44). Elective fees differed substantially—depending on the elective destinations and time point. Median elective fees were highest in the United States of America (€ 1875 for a 4-week elective between 2018–2020), followed by the Republic of South Africa (€ 400) and Australia (€ 378). The data also suggests an increasing trend for elective fees, particularly in the United States.

**Conclusions:**

Rising fees warrant consideration and a discussion about the feasibility of reciprocity and the bidirectional flow of students in bidirectional exchange programs.

## Background

International medical electives are a popular component of the curriculum of many medical schools and enjoy huge popularity among medical students [1, 2]. Abroad electives are nowadays a common part of the student’s final year experience and considered a necessity to provide global health training, and to facilitate international clinical experiences [3, 4].

Globalization has given medical students the opportunity to pursue electives abroad, which may enhance the long-term socialization of medical professionals and foster cross-cultural exchange in a globalized world [5–9]. Electives also allow students for a global view on health problems of current times in a world where “the separation between domestic and international health problems is no longer useful” (Dr. Brundtland, Director General of the World Health Organization 1998–2003) [10].

There are undeniable benefits to practicing medicine in a foreign and unfamiliar setting, and former students frequently describe international medical electives as one of the highlights of their time at medical school [11].

International electives are also recognized as high-impact practice in clinical education [12]. Students often value their abroad experience to enhance their clinical skills and to aid in the understanding of different healthcare systems [13]. Electives may additionally provide students more responsibility and experience within diverse clinical environments [14, 15].

Of note, there are several barriers and challenges that students face when working outside of their comfort zone [16]. The biggest challenge in the past four years was most likely to secure an international elective placement after all [14]. Large scale lockdown measures, uncertainty posed by virus variants and unpredictable travel restrictions continued to stand in the way of reliably organizing an international elective during the COVID-19 pandemic. In addition to that, many medical schools did not offer rotations for visiting students, and switched face-to-face campus-based teaching to virtual platforms during the pandemic [17–19].

**Fig. 1:**
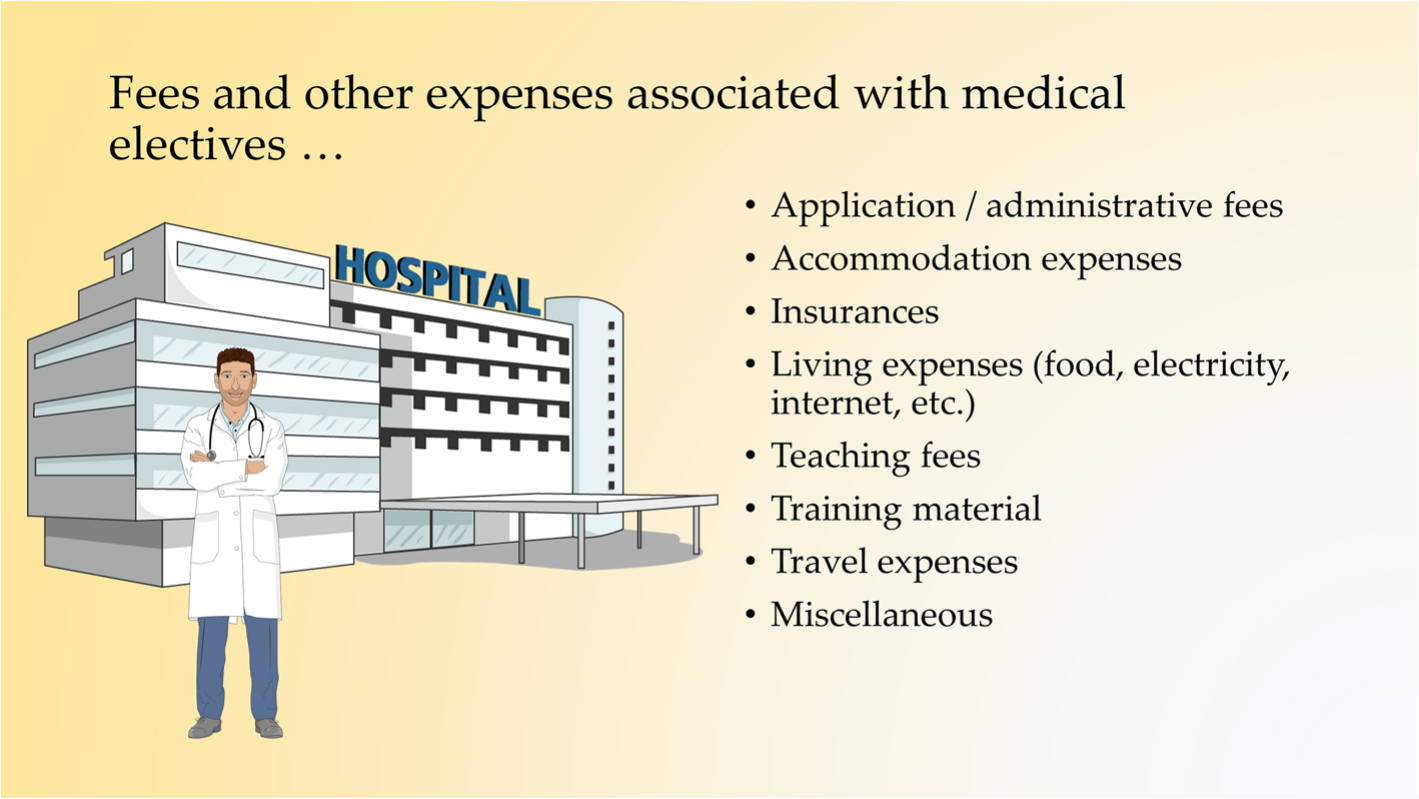
Fees and expenses associated with medical electives – an overview. Based on [22]. Modified from Servier Medical Art database by Servier (https://smart.servier.com/; Creative Commons 3.0)

Moreover, many institutions and universities, particularly in the United States of America (US), have stringent application requirements and long application processes [20]. Another often underrated barrier worth consideration includes elective fees and other costs related to medical electives (Fig. 1) [20–22].

High application fees (in some cases up to $1,000 per application) [20] and teaching fees (in some cases more than $4,000 per month) may not be ignored [21]. Based on the authors’ personal experience and our research in the field of international undergraduate electives [22], these elective fees changed substantially within the last years. In some countries, we observed skyrocketing elective fees within the last decade, with some universities charging more than $1000 per elective week. These costs are believed to cover both tuition and administration expense. While about two decades ago many universities did not charge incoming elective students at all, this has changed substantially over the years. Free elective placements in countries such as Australia, Canada, the United Kingdom (UK), New Zealand or the US are nowadays scarce and hospitals often receive more applications than elective placements available [22].

Of note, international elective fees, price fluctuations and trends during the last years have never been subject to dedicated scientific investigations. We sought to address this gap in the literature, and explored trends in international elective fees within the last 15 years in some of the most popular elective destinations, including the US, the UK, the Republic of South Africa (RSA) and Australia. Here, we investigated the hypothesis that elective fees for international medical electives are subject to a constant increase.

## Methods

This article is part of a larger series on international medical electives and the methods have been described in great detail elsewhere [8, 23–26]. In brief, we extracted data from the two most popular German online databases cataloguing international elective testimonies. The analysis included short-term elective reports (retrieved from the “Famulatur-ranking” database (www.famulaturranking.de) as well as long-term elective reports (retrieved from the PJ-Ranking database (www.pj-ranking.de). Both databases are in German language and mainly used by students based in Germany, Switzerland and Austria [22, 24]. Upon completion of an elective, German students often rate their experience and upload a self-written report (which includes core information on duration, country and home institution as well as the elective institution and elective discipline) to the aforementioned open-access databases. This is a voluntary task and thus reports usually differ in style and length. A minimum set of information covering the aforementioned variables is necessary to upload the report. All reports are completely anonymous and not traceable to the author.

Two independent reviewers extracted data in January 2022 (KB and MAS). Data was captured by convenience sampling.

For our analysis, we particularly focused on abroad electives in the US, the UK, Australia and New Zealand (combined in one group), Ireland, and the RSA. International clinical electives at a renowned institution in the Anglo-American countries (especially in the United States or Australia) are highly popular among medical students based in Germany, and considered a strong career boost for those students aiming for an academic career [24].

In a first step, all eligible reports were screened individually. We extracted key report data to a Microsoft Excel file. This included elective type, elective destination (country, city and hospital), elective duration, and the elective fee. The elective fee encompassed both application and tuition fees. Afterwards, we excluded all duplicates of identical reports that were uploaded more than once. Two reviewers independently extrapolated the data (KB and MAS). Only complete reports that included all the aforementioned variables were considered for the present analysis.

Afterwards, we excluded elective testimonies that did not specify an elective fee. Moreover, we also excluded electives that were exempted from a fee, for example international electives as part of a bi-lateral (medical elective) exchange program or electives that fell under a memorandum of understanding between multilateral parties.

We analyzed all elective reports from 2006 until 2020. International electives in 2021 and 2022 were not considered for the very low number of available reports of abroad electives during the pandemic, and due to the unprecedented elective conditions during the COVID-19 pandemic (e.g. restricted global traveling and large scale elective cancellations) [26–28]. For our trend analysis, and in light of the available report numbers, we grouped electives in 3-year blocks (e.g. 2020–2018; 2017–2015, 2014–2012, 2011–2009, 2008–2006).

Students reported electives fees in different currencies (e.g. in €, £, R or US$). Fees were edited to standardize the reported amount to number of € per one elective of 4 weeks. We obtained historic (year-specific) exchange rates from a widely used currency converter (https://fxtop.com/). The latter provides means of the respective exchange rate per year (e.g. the mean EUR/USD conversion rate for 2008, 2009, etc.)

Initially, we performed a general elective fee trend analysis (including all countries). In addition to that, we performed a trend analysis for each country to investigate potential differences. For the statistical analysis, we used SPSS Statistics (IBM Corp. Released 2020. IBM SPSS Statistics for Windows, Version 27.0. Armonk, NY: IBM Corp). We analyzed the available data for normal distribution. As data was not normally distributed, results were shown as medians (interquartile range). We performed Kruskal–Wallis-tests for an exploratory comparison of elective fee differences by time block and differences by elective destination. Due to an expected impact of destination on elective fees, subgroups analyses of different countries were performed in the same way. A *p*-value of less than 0.05 was used to determine statistical significance.

## Results

**Fig. 2:**
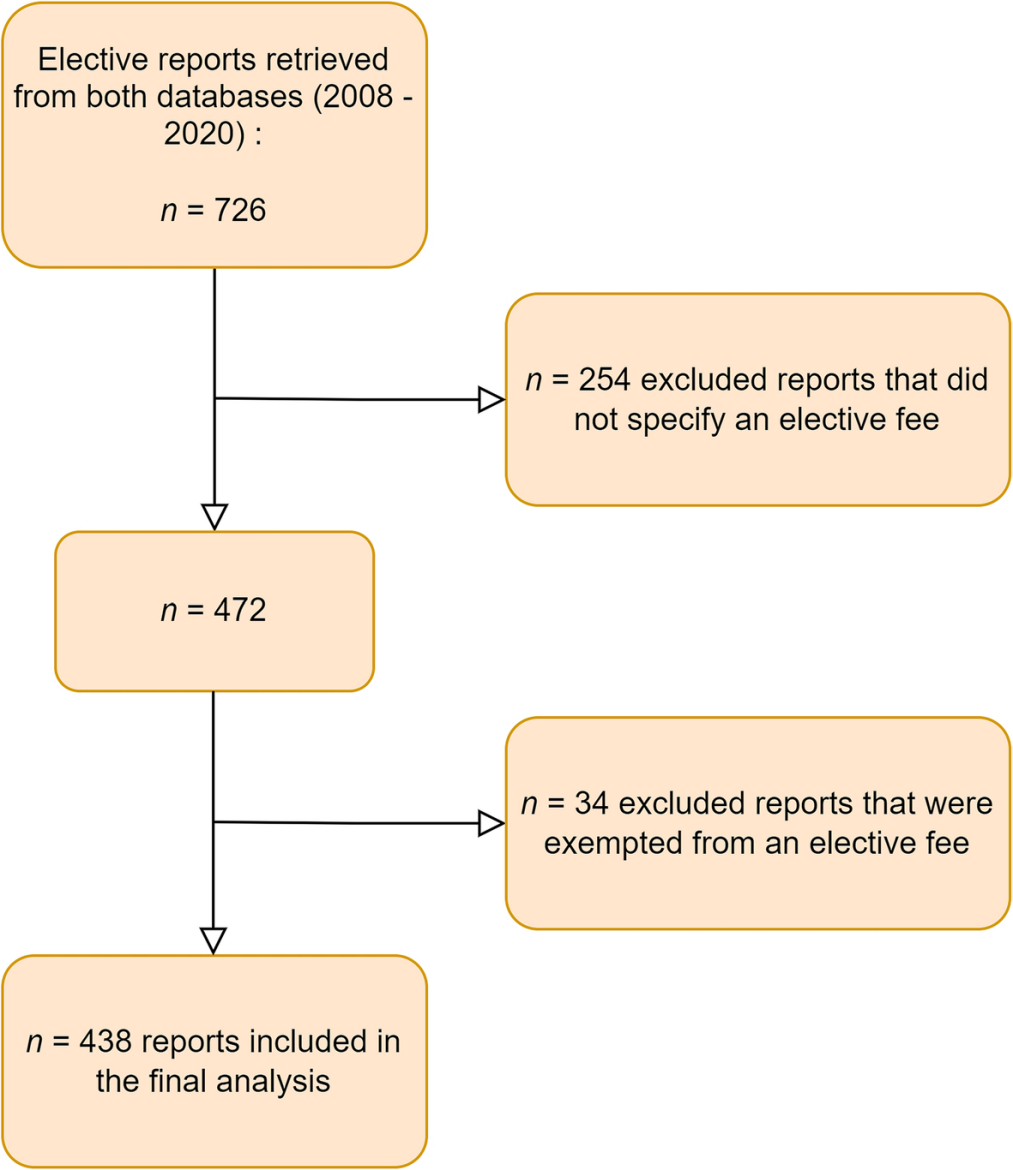
Elective reports inclusion flowchart. *n* = number of reports

**Fig. 3:**
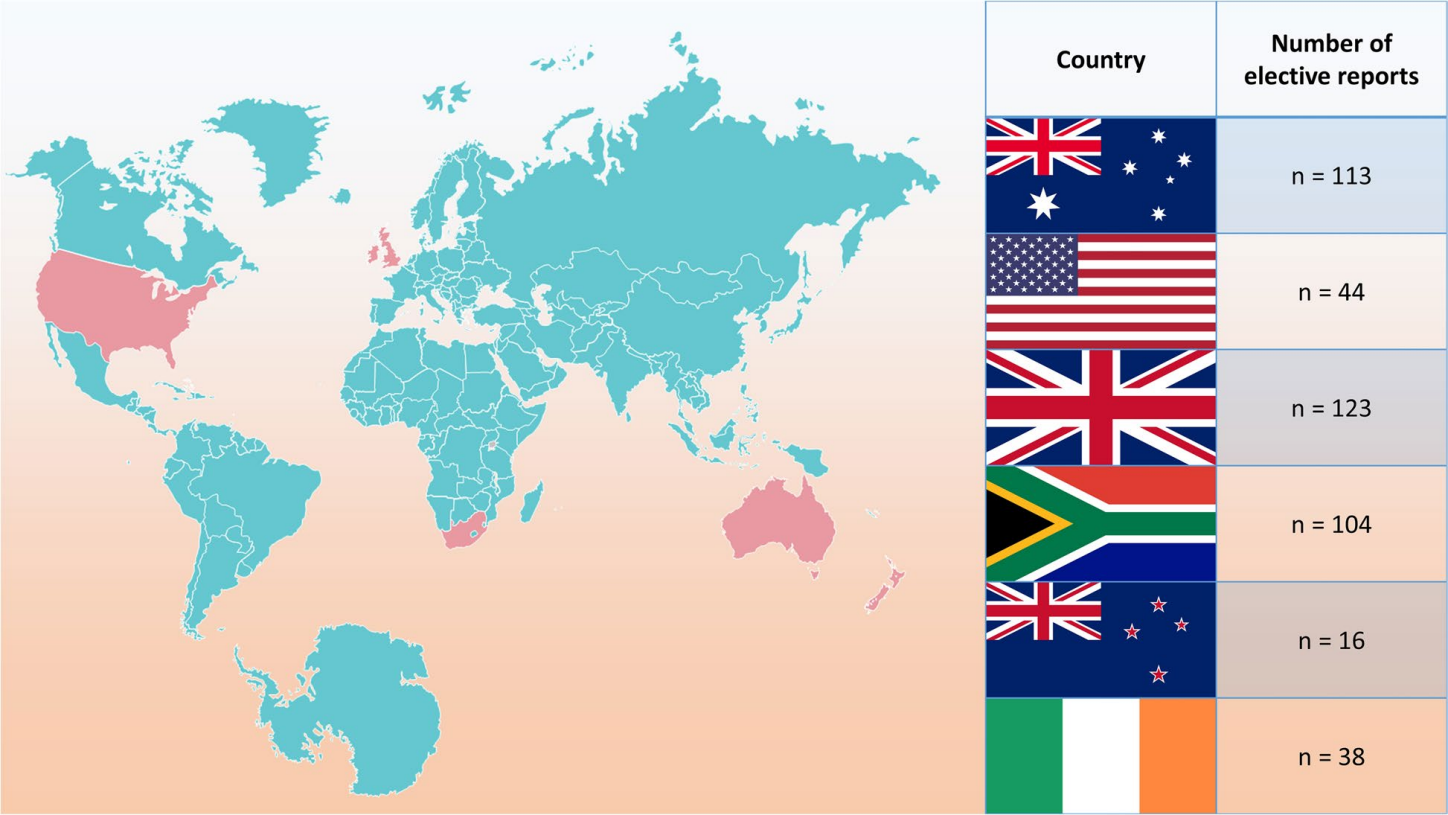
Number of total elective reports per country – an overview. Modified from Servier Medical Art database by Servier (https://smart.servier.com/, Creative Commons 3.0)

We identified 726 overseas elective reports (for the US, Australia and New Zealand, the UK, Ireland and the RSA) that were uploaded to both databases between 2006 and 2020 (see inclusion flow chart Fig. 2). After removal of all reports not meeting the inclusion criteria, a total of 438 elective reports remained eligible for our final analysis. Figure 3 shows the total number of reports per country in our analysis.

Fifty-six short-term (overseas) elective reports were uploaded to “Famulatur-ranking” and another 382 long-term (abroad) elective reports were uploaded to “PJ-ranking”. On average, 31 elective reports were uploaded for all countries combined per year between 2006 and 2019. This numbers dropped substantially in 2020, with only 9 reports uploaded in total. Table 1 displays the number of elective reports per 3 years for each examined country during the investigated time frame. The UK (28.1% of reports), Australia (25.80% of reports) and the RSA (23.74% of total reports) were the most selected elective destinations in our sample, whereas New Zealand accounted for only 3.65% of total reports (Table 1).

**Table 1:** Number of elective reports per 3 years by country: an overview

Year	United States of America	Australia	New Zealand	Ireland	United Kingdom	South Africa
2020—2018	*n* = 6	*n* = 12	*n* = 6	*n* = 14	*n* = 28	*n* = 31
2017—2015	*n* = 5	*n* = 31	*n* = 2	*n* = 14	*n* = 34	*n* = 21
2014—2012	*n* = 4	*n* = 25	*n* = 3	*n* = 4	*n* = 30	*n* = 18
2011—2009	*n* = 22	*n* = 32	*n* = 4	*n* = 5	*n* = 25	*n* = 24
2008—2006	*n* = 7	*n* = 13	*n* = 1	*n* = 1	*n* = 6	*n* = 10

*n* number of observations

Table 2 displays the median elective fees per each 3-year block for the examined country during the investigated time frame. Students spent, on average, 364.15€ on elective fees for a 4-week elective (all countries combined, 2006–2020).

**Table 2:** Elective fees in Euro per 3 years by country: an overview

Year	United States of America	Australia	New Zealand	Ireland	United Kingdom	South Africa
2020—2018	1875 (3948)	378 (151.50)	267.5 (117.06)	150 (0.0)	224 (347.75)	400 (450)
2017—2015	1500 (3087.50)	402 (198)	876 (^a^)	150 (12.50)	140 (272.13)	300 (125)
2014—2012	493.75 (1234.38)	400 (204.40)	300 (^a^)	125 (50)	123 (74.75)	362.75 (375)
2011—2009	360 (352.80)	250 (163.75)	237.50 (483.25)	100 (33.75)	115 (353.25)	238 (558.50)
2008—2006	357 (782.40)	125 (271.50)	50^+^ (^a^)	100^+^ (^a^)	146.25 (429.98)	250 (179.30)

^a^Interquartile range not calculable due to low sample-size; +  = absolute value (*n* = 1)

**Fig. 4:**
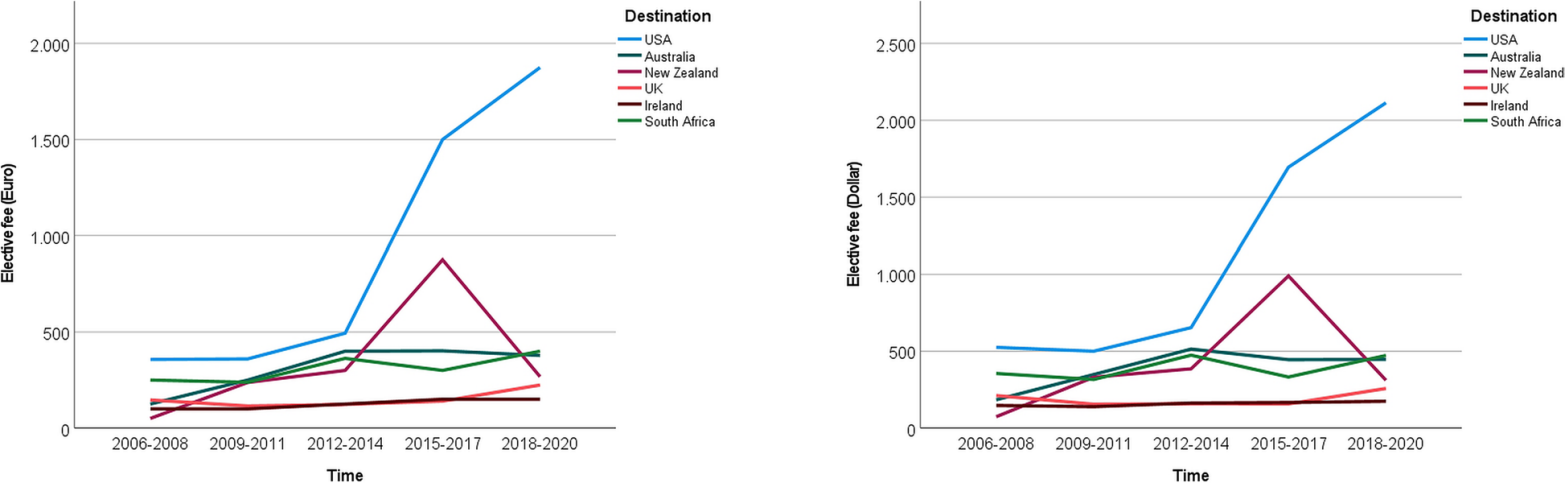
Elective fees – a trend analysis. Trend analysis by country and year; elective fees are shown in Euro (left panel) and US Dollars (right panel)

Comparison of elective fees (without consideration of destination) revealed no statistically significant differences between the examined time blocks, but distinct destination-related differences were found (Fig. 3). Electives in the US were significantly costlier than electives in other countries (*p* < 0.001). The median elective fee for a 4-week elective between 2018 and 2020 in the United States was 1875 Euros. The elective fee in the US increased significantly over time (*p* = 0.45), as shown by our trend analysis in Fig. 4. Electives in other countries were less costly (e.g., the European destinations). Median elective fees continuously increased between 2006–2008 and 2015–2017 in Australia. We also observed a comparable overall trend for South Africa.

## Discussion

Scientific literature about international medical electives is generally scarce [29], and no study has specifically investigated elective fees for abroad electives within the last decades. We sought to address this gap in the literature, and explored trends in abroad elective fees within the last 15 years in some of the most popular elective destinations among students from the German-speaking countries. Our results demonstrated that these fees differed substantially depending on the elective destination and time point. Median elective fees in South Africa and Australia were higher as compared to the UK and Ireland. Median elective fees were highest in the United States of America, where they also increased substantially over the last decade.

There are undeniable benefits to practicing medicine in a foreign and unfamiliar setting [11, 30], and international electives also play a key role in global health education [31, 32]. The priority of global health is to improve health and to achieve equity in health for all people worldwide in a globalizing world [33]. A popular definition of global health states that “*global health emphasizes transnational health issues, determinants, and solutions; involves many disciplines within and beyond the health sciences and promotes interdisciplinary collaboration; and is a synthesis of population-based prevention with individual-level clinical care*” [33].

Healthcare is increasingly globalizing, and so are medical problems and disease patterns. As such, some experts highlighted that “the separation between domestic and international health problems is no longer useful” (former General Director of the World Health Organization).

Medical schools responded to the increasing need for practicing healthcare from a “global point of view” and there has been a growing emphasis on globally-relevant health professions education [32]. Interest in global health among medical students worldwide is measurably increasing and medical students worldwide travel abroad to gain a better and deeper understanding of international and global health problems [32].

International abroad electives, however, are not without costs and require extensive planning. High application and teaching fees associated with international electives are increasingly common [21, 22], and may constitute an insurmountable obstacle for students from resource-poor settings.

As shown in Fig. 1, there are many expenses associated with international electives, and elective fees (covering both the application and tuition) are just one component of the total-cost calculation for an abroad adventure. It is conceivable that elective fees in a range of several thousand dollars for a 4-week elective may discourage international students with limited financial resources from applying. Although waiver programs exist occasionally, one may not forget that there are other costs to consider (Fig. 1). As such, only students with a certain financial background may nowadays be blessed with the opportunity to engage in international electives.

The substantial increase in elective fees observed in some countries (e.g. for the US in our sample) may worsen this situation. Our data thus raise the question whether international electives are more and more becoming a commodity that is merely available to privileged students? Hypothetically speaking, increasing elective fees could intensify the existing North–South gap that has been previously described by Hanson et al. and other authors [34, 35]. Still, the majority of electives involve travel from northern, higher-income countries to southern, lower-income countries [35]. Increasing elective fees could be an important contributor to this phenomenon, and may even worsen the situation.

This unidirectional flow of students from economically advantaged to resource-poor nations, in turn [36], may lead to increasing disparities and inequity in medical education worldwide. Efforts to expand medical training in resource-constrained settings and to enable students from these backgrounds to partake in international electives in well-situated countries are thus warranted [37]. One potential solution could be the introduction or expansion of existing international bi-directional elective exchange programs. Ghanaian medical students who participated in a U.S.-based elective, for instance, reported valuable insights and development of skills and knowledge that shaped future decisions regarding their training and career choices [36]. However, previous studies demonstrated that particularly US institutions often do not host students from international sites [38]. Rohrbaugh et al. emphasized that although the majority of US institutional partners do accept international students, more than one-fifth of US schools with a structured global health program did not accept students from their international collaborators in return.

Programs focusing on reciprocity in general and the bidirectional flow of students in particular should thus be considered essential to develop sustainable and balanced partnerships. Else, students from low-income countries may not be able to stem the costs for a rotation in a high-income country, such as the US.

Apart from elective fees, travel expenses can reach more than $1000 per elective and housing and food may add another $2000 per month of elective experience [38]. In light of these considerable expenses, many students from low-income countries are unlikely to overcome such barriers without assistance. Rohrbaugh et al. discussed potential solutions to this, and highlighted that many fees for visiting students could potentially be mitigated. An example is the fee for an English language proficiency test, which could be assessed quicker and cheaper via a teleconference instead of a certified but often expensive standardized test. This is just one example highlighting potential ways to reduce the financial burden for incoming students. Whether feasible in practice (and with regard to patient safety) is another question that needs to be addressed in future studies. Nevertheless, this example emphasizes the need for creative solutions to enable a bidirectional flow of students in international global health programs. This could also include incentives to host elective students in the university / hospital campus accommodation for a reduced fee. Said measure would reduce the imposed financial burden on visiting students while concomitantly ensuring safe accommodation.

While a detailed discussion of other potential options to ensure this might be beyond the scope of this paper, the authors wish to emphasize the importance of discussing about this topic after all. Elective fees have been very rarely made subject to a scientific discussion, and our data highlight that it is now more than necessary than ever before to speak about this (inconvenient) topic.

From a more general point of view, global health programs and interventions stand to generate significant advantages for donor and recipient countries when appropriately designed, targeted, and delivered [39]. With regard to medical electives, however, it seems that high elective fees could prevent this. As discussed earlier, medical students from resource-poor countries may be excluded from partaking in global electives due to a lack of reciprocity. Disproportionate travel and elective opportunities may represent important barriers to truly practicing “global health” in a globalizing world. From a medical elective point of view, globalizing healthcare and health education also requires a discussion about globalizing medical electives.

Although subject to bias and several limitations (as discussed below), our data also suggested that electives fees have the tendency to increase. We believe that the momentum to tackle this development has come now, in light of the many elective programs that have been placed on hold during the COVID-19 pandemic [25]. Now that international global health programs gradually re-open, students will be automatically confronted with this topic. Medical schools and elective planners may contribute to global health equity by coming up with (economically) feasible and creative solutions that tackle the aforementioned developments. Future research should target such strategies and explore which strategies are most suitable to reach that goal.

### Limitations and strengths

Our analysis has weaknesses and strengths that are worth mentioning. We present a large dataset including more than 438 elective reports. To the best of our knowledge, we also present the first detailed analysis of international electives fees in the literature. Our findings are of high translational value, and highlight a potential problem that has so far received little attention. Our data could be of high value for medical institutions, universities, elective coordinators and other stakeholders involved in international electives.

At the same time, the present analysis has several limitations. We carried out no personal interviews and our analysis is based solely on written reports from two open-access databases. As such, the analysis may be subject to bias. Direct elective testimonies based on qualitative (semi-structured) interviews may have been superior to our approach, yet both interrogated databases do not routinely save student contact data. In addition to that, we acknowledge that we present data from a convenience sample, which may not be representative of the general (German) medical elective landscape. Yet, to the best of our knowledge, we present data from two of the largest elective databases existent, and comparable case numbers are rare to find in the field of elective research [24].

We also acknowledge that our study did not account for other expenses that students faced when undertaking an elective (e.g. housing or living expenses). Said data would have been most interesting to add but was largely unavailable. Moreover, we had no information on lost earnings by being overseas. Such data would have allowed for a better sense of the total extent of the elective fee issue.

## Conclusion

To the best of our knowledge, we present the first study investigating fees for international medial electives during the past 15 years. We observed substantially differing fees (depending on destination and time point) for a 4-week abroad elective. Recent trends and rising fees (particularly in the US) warrant consideration and a discussion about the feasibility of reciprocity and the bidirectional flow of students in bidirectional exchange programs. While subject to limitations, our data highlight the need for an urgent discussion about the affordability of electives in high income countries. The momentum for this is given right now with regard to the many international elective programs on hold during the COVID-19 pandemic.

## Data Availability

All data associated with this paper will be made available upon reasonable request which should be directed to Maximilian Andreas Storz. Data may also be obtained from www.pj-ranking.de and https://www.famulatur-ranking.de/page/start/.

## Abbreviations

RSA: Republic of South Africa
UK: United Kingdom
US: United States of America
